# Alpha Neurofeedback Has a Positive Effect for Participants Who Are Unable to Sustain Their Alpha Activity

**DOI:** 10.1523/ENEURO.0498-18.2019

**Published:** 2019-08-22

**Authors:** Ankan Biswas, Supratim Ray

**Affiliations:** Centre for Neuroscience, Indian Institute of Science, Bangalore 560012, India

**Keywords:** alpha rhythm, EEG, neurofeedback

## Abstract

Alpha rhythm (8–13 Hz) is linked to relaxed mental state in humans. Earlier reports have shown that individuals can increase their alpha power if provided with a valid feedback, compared to controls who are provided invalid feedback. However, these results remain controversial, partly because controls may be in a different behavioral state, making it difficult to directly compare their alpha power with the valid group. We here address this issue by using an experimental paradigm in which an invalid feedback is given on a fraction of trials, such that both valid and invalid conditions can be obtained from the same participant. Using electroencephalography (EEG), we recorded alpha power from the occipital area from 24 humans (nine females) and played a feedback tone which could be valid (tone frequency proportional to alpha power), invalid (tone sequence from a previous valid trial; participants were unaware of this condition), or neutral (constant tone frequency). We found that during eyes closed-state, neurofeedback did not enhance alpha activity beyond pre-trained state within the experimental duration, probably because of saturation of alpha rhythmicity. However, for participants whose alpha power decreased over time within a trial, valid feedback helped them to sustain alpha more than invalid feedback. Further, alpha increase showed a weak negative correlation with their self-reported attentional load but was uncorrelated with relaxation levels. Our results reconcile many conflicting reports in the neurofeedback literature, and show that even under most stringent control, valid neurofeedback can help participants who are otherwise unable to sustain their alpha activity.

## Significance Statement

We tested whether providing a real time auditory feedback about the strength of the EEG alpha rhythm helps the participants increase their alpha power. Unlike previous neurofeedback studies that used valid and invalid feedback on different participant groups, we used a design in which valid, invalid and neutral feedback were given to the same participant. We found that for participants whose alpha power reduced over time within a trial, valid feedback helped to sustain the rhythm better than invalid feedback. Further, feedback appeared to be more useful for participants who did not attend the tone. These findings can be used to better screen and design neurofeedback training paradigm, which is now used to treat patients suffering from anxiety and depression.

## Introduction

In EEG signals, a brain rhythm in the frequency range between 8 and 13 Hz, called alpha, is prominently observed in the occipital area of many individuals, especially during an awake and relaxed state with eyes closed (Berger, 1929; Adrian and Matthews, 1934). Although alpha was traditionally believed to be an idling rhythm, recent studies have linked alpha rhythm with high-level cognitive mechanisms such as attention (Kelly et al., 2006a; Klimesch et al., 2007), information retrieval (Klimesch, 2012), and creativity (Fink and Benedek, 2014). Therefore, it has been suggested that learning to control the alpha activity may have a positive effect on the mental state (Escolano et al., 2011; Zoefel et al., 2011; Klimesch, 2012; Nan et al., 2012).

Alpha Neurofeedback involves providing individuals a real-time feedback about their alpha power (Kamiya, 1969, 2011), which is typically provided by a tone (Kamiya, 1969; Plotkin, 1978; Dempster and Vernon, 2009; van Boxtel et al., 2012), or occasionally by a visual signal (Brown, 1970; Dempster and Vernon, 2009; Ros et al., 2010, 2013). Early neurofeedback studies reported that participants could learn to enhance their alpha activity with the aid of neurofeedback training (Kamiya, 1969; Brown, 1970; Nowlis and Kamiya, 1970; Hord and Barber, 1971; Hardt and Kamiya, 1976), which could further have a beneficial effect on their behavioral state, such as reduction in anxiety (Garrett and Silver, 1976; Hardt and Kamiya, 1978) or sleep need (Regestein et al., 1973). However, these findings were subsequently challenged, because the constitutional, physiological and cognitive-attentional state of the participant could vary during training, and that itself could change alpha power (for review, see Lynch and Paskewitz, 1971; Plotkin and Rice, 1981; Rice and Blanchard, 1982). For example, participants may be anxious/attentive during the beginning because of an unfamiliar setting and may get more relaxed during the training. This alone could increase alpha power over time, irrespective of feedback.

One way to address this concern is to have a “control” group to which invalid or no feedback is provided. Early studies that employed such controls gave conflicting results, with some studies showing an increase in alpha even with no/invalid feedback (Strayer et al., 1973; Lynch et al., 1974; Lindholm and Lowry, 1978), while others showing no increase without valid feedback (Beatty, 1971, 1972). Some studies attributed this discrepancy to methodological differences (Paskewitz and Orne, 1973; Walsh, 1974; Hardt and Kamiya, 1976; Ancoli and Kamiya, 1978; Vernon et al., 2009). Others have suggested that even this design is not sufficient (Rogala et al., 2016; Biswas and Ray, 2017), since the behavioral state of the control and contingent groups may be different. For example, the control group may stop paying attention to the feedback if they realize that it is not helping them. Further, small effects may not be observed due to large inter-participant variability in alpha power across the contingent and control groups (Haegens et al., 2014). To address these concerns, a design is needed in which each participant could potentially be his/her own control (Biswas and Ray, 2017).

To address this, we designed an experiment in which we provided invalid feedback (representing alpha activity from a trial in a previous block) to the participants in 25% of trials, along with valid (50%) and no feedback (25%). The participants were completely unaware about the invalid trials, and therefore the behavioral conditions (for example, amount of attention paid to the feedback tone) were identical to the valid case. We then investigated whether valid feedback had a stronger effect on alpha power than invalid feedback. Further, in our design the participants were free to either use or ignore the feedback, which allowed us to study the correlation between enhancement of alpha power with subjective attention and relaxation levels, which participants provided after the task.

## Materials and Methods

### Participants

Twenty-four healthy volunteers (mean age: 23.9 years, females: 9) participated in the study. The protocol used for EEG recording was approved by the Institutional Human Ethics Committee of the Indian Institute of Science (IISc). Before conducting the experiment, the participants were briefed about the experimental procedures (see Experimental paradigm) and the risks involved, after which written informed consent was obtained. Participants were requested to sit comfortably in front of a computer monitor and to avoid any unnecessary movements during the experiment.

### Experimental paradigm

**Figure 1.**
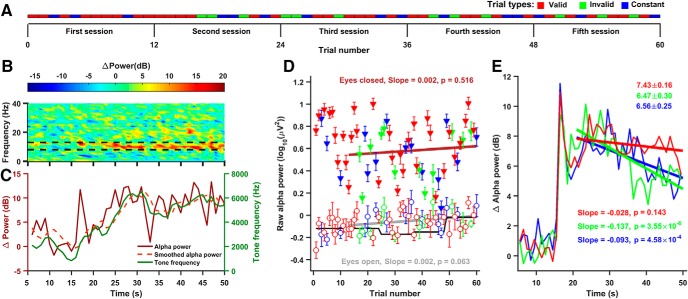
Effect of neurofeedback training on alpha power in a representative participant. ***A***, Details of the experimental paradigm. Three types of trials, namely valid (red), invalid (green), and constant (blue) were presented for five sessions, each consisting of 12 trials. Each trial was 50 s long. Each block started with a calibration stage (data not shown). The first session, in which invalid trials were not presented, was not used for analysis. ***B***, Time-frequency spectogram of a single valid trial showing change in power from baseline (computed during the calibration stage). Broken lines at 8 and 13 Hz indicate the alpha range. ***C***, Change in instantaneous alpha power for the same trial as in ***B*** (brown trace; left *y*-axis). Dotted orange line depicts alpha power smoothed by averaging across the previous 5 s, which was used to set the frequency of the feedback tone (green trace; right *y*-axis). ***D***, Raw alpha power versus trial number during calibration (thick black line; same value for each block of 12 trials), eyes open (open circles), and eyes closed state (solid triangles). Regression lines between raw alpha power versus trial number (13–60) are shown for eyes open (gray trace) and eyes closed states (brown trace). Corresponding slopes and *p* values are indicated in the panel in respective colors. Error bar indicates SEM. ***E***, Change in alpha power with respect to time for three types of trials: valid (red), invalid (green), and constant (blue), averaged over trials 13–60 (24 valid, 12 invalid, and 12 constant trials). Regression lines plotted between mean change in alpha power and time (21–50 s) are also shown in corresponding colors. Average change in power in decibels (between 21 and 50 s) ±SEM for the three types trials are indicated at top right corner (in corresponding colors). Slopes of the regression lines along with their *p* values are indicated at bottom right corner.

The experiment was divided into five sessions. Each session consisted of a calibration stage (15 s), followed by 12 trials of 50 s each (Fig. 1*A*). There were three types of trials: valid, invalid, and neutral/constant. During valid trials, the frequency of the feedback tone was directly proportional to the change in alpha power from baseline (computed during the calibration phase). During invalid trials, which were presented from the second session onwards, one of the valid trials from the first session was chosen and the tone sequence for that session was presented. For constant trials, the frequency of the feedback tone was kept constant throughout the trial. The first session (termed “pre-training” phase because the subjects were naive to the task) consisted of three constant and nine valid trials. From the second session onwards, three invalid trials were presented in each session, along with three constant and six valid trials. The participants were not informed about the invalid trials, so for them, the trial composition was 25% constant and 75% valid for each of the five sessions (the actual composition from second session onwards was 50% valid, 25% invalid, and 25% constant). The entire trial sequence was generated pseudo-randomly for each participant at the beginning of the experiment. Note that the first session was not used for analysis because it had no invalid trials and the participants were getting accustomed to the task during this session. However, including the first session data for the analysis yielded very similar results (data not shown).

Each session started with a calibration process, in which participants were asked to keep their eyes open without blinking. The calibration process yielded a “baseline” value of the alpha power (average alpha power between 6 and 15 s), which was used for calibrating the pitch of the feedback tone for that session. The calibration process was occasionally repeated if the participants blinked or there was any movement artifact (assessed by manual inspection of the time-frequency spectrum of the EEG signal that showed a broadband response due to such artifacts), although this happened rarely. We did not implement any online artifact rejection. During the neurofeedback training, participants had their eyes closed, so no significant artifacts were observed related to eye movement/blink.

During each trial, for the first 15 s, participants were asked to keep their eyes open. From 6th second onwards, a tone was played whose frequency was modulated in three different ways depending on the trial type, as described above. Fifteen seconds after the trial onset, a message was displayed on the monitor screen instructing participants to close their eyes and relax as much as possible. The participants were instructed to try to maximize a “performance score,” which reflected the average change in alpha power from the baseline power (measured during calibration time) in the interval between 21 and 50 s after trial onset and was displayed at the end of the trial on the monitor screen. This score reflected the true change in power, irrespective of the trial type. Once the trial ended, the tone stopped, and the experimenter asked the participant to open his/her eyes and view their performance score. There was no fixed inter-trial interval; the participants simply indicated by a hand gesture to the experimenter whenever they wanted to start the next trial. The total duration of the experiment was ∼1.5 h.

Importantly, participants were told that the pitch of the feedback tone in non-constant trials was proportional to the relaxation score, but they were not instructed to explicitly pay attention to the feedback tone. Specifically, they were told that they had the liberty to use the feedback tone to improve performance but could also ignore the feedback tone if they felt it was distracting and was not aiding them in increasing the performance score. Indeed, different participants used different strategies, as revealed by their responses to a questionnaire presented at the completion of all the sessions.

### Questionnaire

After the experiment, all the participants were required to fill up a questionnaire consisting of the following four questions. (1) Was the task relaxing? (relaxation score: 1, not at all relaxing; 10, very relaxing). (2) Was the tone acting as a source of disturbance? (distraction score: 1, not disturbing; 10, very disturbing). (3) Were you using the feedback provided by the tone? (attention score: 1, ignored the tone completely; 10, paid attention to the tone as much as possible). (4) Which method or technique were you using to relax?

Because we asked the participants to fill out the questionnaire only at the end of the experiment, this single evaluation might have been biased by the most recent and vivid experience of the last session only. This issue could have been partially addressed if we had asked them to provide a response at the end of each session (or even perhaps each trial). However, answering questions after each session could have disrupted the continuity of the training/learning and would have increased the duration of the study. Also, we were more interested in the overall effect of neurofeedback that was experienced for the entire duration of the study.

### EEG setup and data acquisition

EEG signals were recorded from all the participants using Brain Amp DC EEG acquisition system (Brain Amp DC, Brain Products GmbH). Five electrodes were placed on the occipital region (PO3, O1, O3, O2, PO4) following international 10–20 standard reference scheme. FCz was used as a reference electrode. Impedance value was kept <10 kΩ for all the electrodes. Raw EEG signal was sampled at 500 Hz, filtered between 0.016 Hz (first-order filter) and 250 Hz (fifth-order Butterworth filter) and was digitalized at 16-bit resolution (0.1 μV/bit).

### Real-time neurofeedback system design

The feedback system was developed using custom written codes in MATLAB (MathWorks Inc., RRID: SCR_001622) and standard socket programming. TCP/UDP/IP MATLAB toolbox (version 2.0.6, GNU General Public License) function *pnet* was used to create a TCP/IP connection between the machine hosting MATLAB and the RDA server of Brain Vision Recorder, which is the proprietary software provided by Brain Products GmbH. Raw data were acquired from the RDA server via TCP/IP protocol into the system port where MATLAB was running. Once the number of data points in the port matched the sampling frequency at which EEG data were acquired by the Brain Vision Recorder (1 s of data), it was further processed in MATLAB for power estimation.

Power of this 1-s-long signal was estimated using multitaper method using a single taper, implemented in the Chronux package (Bokil et al., 2010), yielding a frequency resolution of 1 Hz. Power was first averaged across electrodes, and then averaged over the alpha range (8–13 Hz) to get alpha power. Because the alpha power varied considerably over time, we took the average power over previous 5 s for generating the feedback tone (Fig. 1*C*, dotted line). Consequently, the feedback tone could be provided only from 6th second onwards. We calculated the change in alpha power as follows:

(1) $$\Delta {\text{P}}_{\text{α}}(\text{t})=10\times \log_{10}\frac{{\text{P}}_{\text{e}}(\text{t})}{{\text{P}}_{\text{c}}},$$

where ΔP_α_ denotes the change in alpha power in decibel calculated at time t; P_e_ is the mean alpha power over a 5-s interval preceding t, and P_c_ is the mean alpha power during the calibration period (taken only once per session). The frequency of the feedback tone (Fs, in Hz), played to the participants using a speaker located in front of them, was calculated according to the following equation:

(2) $${\text{F}}_{\text{s}}\left(\text{t}\right)=1000+\Delta {\text{P}}_{\text{α}}\left(\text{t}\right)\times 500.$$

Note that for estimating alpha power, we did not bandpass filter the EEG data, but instead used multitaper method and averaged the power in the alpha band instead. Further, this analysis was performed separately at each second of data, and hence there was no overlap in the analysis windows (although we averaged power estimates over the previous 5 s for generating the feedback tone). Once the tone frequency was estimated (Eq. 2), the tone was generated for 1 s. The delay between subsequent feedback tone signals was limited to the computational time to perform these analyses once data were collected for that second. Behaviorally, successive feedback tones appeared almost instantaneously with no gap, suggesting that the computation time was negligible.

### Statistical analysis

All statistical analyses were performed in MATLAB (MathWorks Inc.). One-sample two-tailed *t* test was used at significance level α = 0.05 to check whether the slopes of the regression lines were significantly different from zero. To check whether mean alpha power duirng valid trials was significnaly different compared to invalid trials, two-sample one-tailed *t* test was performed at the significance level α = 0.05 assuming unequal variances. Bonferroni correction was done to adjust the significance level to α = 0.0021 (0.05/24) whenever multiple comparisons across 24 participants were required.

## Results

We recorded EEG (Brain Amp DC, Brain Products GmbH) from 24 healthy young adults using five active electrodes covering the occipital area. Figure 1*A* shows the experimental paradigm, which consisted of 60 trials of 50 s each, divided into five sessions. An auditory feedback was provided in each trial, which could be valid (red), invalid (green; given only from second session onwards), or a constant tone (blue).

Figure 1*B* shows the change in time-frequency power (in dB) in one valid trial (trial 16), from a baseline power computed in a calibration stage just before the start of the session (in this case, before trial 13). Alpha Power was enhanced by >5 dB as soon as this participant closed eyes (16th second onwards) and remained high until the end of the trial (50th second). Figure 1*C* shows the change in alpha power over time, calculated by averaging the power in the alpha band (between the dotted black lines shown in Fig. 1*B*), which showed a transient peak at the 16th second just when the eyes were closed, and remained high thereafter. This change in alpha power was averaged over a 5-s window (dotted line) and used to set the frequency of the feedback tone (green trace).

Figure 1*D* shows the alpha power for all trials for this participant, during eye closed (21–50 s after stimulus onset; filled triangles) and eye open (6–15 s, open circles) states. The baseline power used for setting the feedback tone frequency (same value for each trial within a session) is shown by a black line, which was comparable to the alpha power during the eyes open state. To test whether feedback training enhanced alpha power over time, we performed linear regression analysis between alpha power and trial number (starting from session 2). For this subject, the slopes were not significant for either eye open or eye closed conditions (eyes open: slope = 0.002 ± 0.001, *t*_(47)_ =1.903, *p* = 0.063; eyes closed: slope = 0.002 ± 0.002, *t*_(47)_ = 0.655, *p* = 0.516). Results were similar when the analysis was restricted to trials of the same type (data not shown).

Across the population of 24 participants, the mean slopes of alpha power versus trial number were 0.002 ± 0.0005 and 0.001 ± 0.0006 for eyes open and eyes closed states, respectively. For eyes open condition, the mean slope was significantly different from zero (*N* = 24, *t*_(23)_ = 2.936, *p* = 0.007). However, the behavior of the participants was not well controlled during this condition; it is possible that they made different strategies during this phase early during the experimental session, which could have lowered their alpha power. Importantly, the slopes were not significantly different from zero (*N* = 24, *t*_(23)_ = 1.594, *p* = 0.125) during the eyes closed condition, during which their behavior was more controlled, and their alpha power was much higher than the eyes open condition. Therefore, during the eyes closed period, neurofeedback training did not significantly enhance alpha power over the entire course of the experiment.

To test whether the type of feedback had any effect on alpha power within a trial, we plotted the change in alpha power from baseline averaged over the three trial types (Fig. 1*E*). For all trial types, a peak was observed as soon as this participant closed the eyes (16th second), which may be the “alpha squeak” effect on eye closure (van Leeuwen et al., 1960). During the later period (after ∼25 s), however, α power decreased for the invalid and constant trials, but remained relatively more elevated for valid trials. To quantify this, we again performed linear regression analysis between change in alpha power and time between 21 and 50 s. For this subject, slopes were significantly negative for invalid and constant trials, but not for valid trials. Consequently, the average change in alpha power was about ∼1 dB larger for valid as compared to invalid trials (7.43 vs ∼6.47 dB). Note that although the invalid condition had the same stimulus statistics and presumably the same behavioral state as the valid one, the alpha power for invalid condition was actually more similar to the constant condition for which the tone was constant and uninformative.

**Figure 2.**
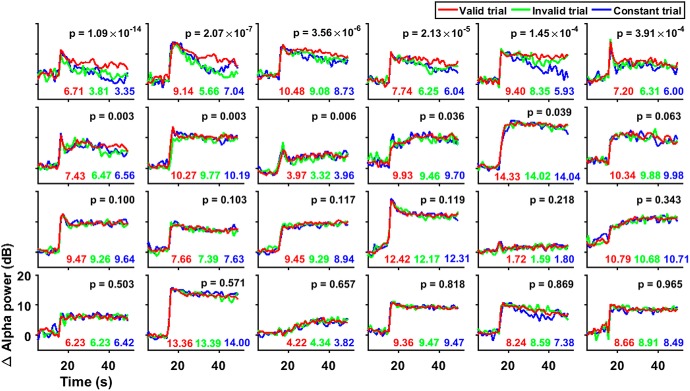
Modulation of alpha power for three types of trials for all participants. Same as Figure 1*E*, separately for each of the 24 participants, in descending order of significance of the difference between change in alpha power between valid and invalid trials (estimated using *t* test and shown in the plots, along with the mean changes in alpha power for the three trial types).

Figure 2 shows the same analysis as shown in Figure 1*E* for all 24 participants, sorted in decreasing order of significance of the difference in mean alpha power (averaged between 21 and 50 s) between valid and invalid conditions. The alpha power between valid and invalid conditions was significantly different for 11 (6 after Bonferroni correction for the number of participants) out of 24 participants, suggesting that the effect of neurofeedback was subtle and not applicable to all participants. Interestingly, like Figure 1*E*, alpha power in the invalid condition was similar to the constant condition for several subjects who showed a positive effect of neurofeedback, although the stimulus and behavioral aspects for invalid condition were matched to the valid condition. This suggests that the evolution of alpha power over time may actually depend on the “usefulness” or “information” provided by the feedback, since both invalid and constant trials were, on average, equally un-informative.

**Figure 3.**
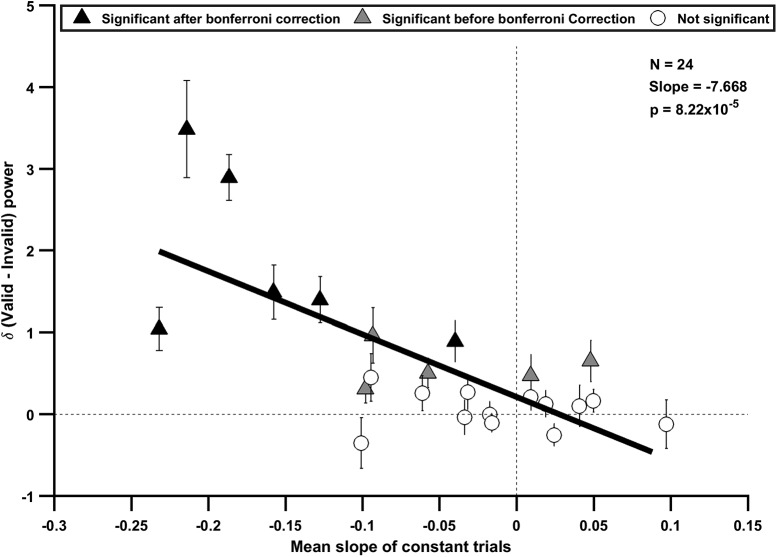
Effect of neurofeedback depends on sustenance of alpha power. Scatterplot shows difference in power between valid and invalid trials (Δ power) and slope for the constant trials for all the 24 participants. Thick black line shows a regression line; slope and *p* values are indicated in the panel. Participants 1–6, for which mean change in alpha power between valid and invalid trials was significant after Bonferroni correction, are indicated using filled black triangles. Participants 7–11, for which the difference was significant before Bonferroni correction, are indicated using gray triangles. Remaining participants are indicated using open circles. Error bar indicates SEM.

A closer look at Figure 2 revealed an interesting trend for the participants who showed a significant effect: for most of these participants, alpha power appeared to decrease over time, suggesting that these participants could not sustain their alpha rhythm. To quantify this effect, for each participant, we plotted the slope of alpha power versus time for the constant trials (Fig. 1*E*, blue line) versus the overall change in alpha power between the valid and invalid conditions (Fig. 3). Indeed, the participants who showed a significant increase in alpha power also tended to have negative slopes, and there was a strong negative correlation between the two variables (slope = –7.668 ± 1.591, *t*_(23)_ = –4.817, *p* = 8.22 × 10^−5^). This suggests that neurofeedback mainly helped participants increase their alpha power who could not otherwise maintain their alpha activity.

**Figure 4.**
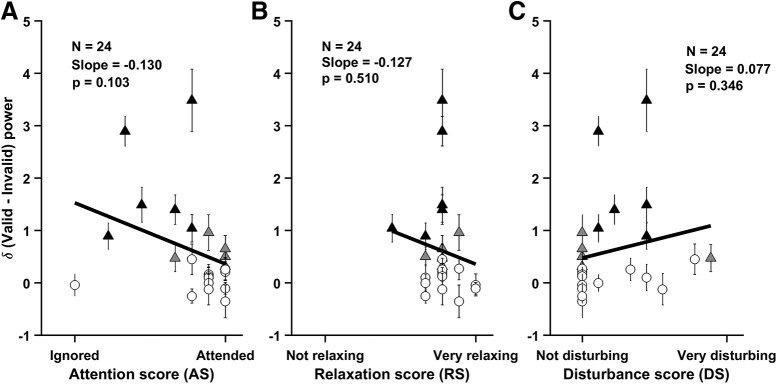
Subjective experience of the neurofeedback training for all participants. Scatterplot showing difference in alpha power between valid and invalid trials with respect to (***A***) feedback scores (FSs), (***B***) relaxation score (RS), and (***C***) disturbance scores (DSs). For each plot, regression line is shown in thick black, and slope and *p* values are indicated in the panel. Same markers as in Figure 3. Error bar indicates SEM.

Finally, we tested whether the change in alpha power was correlated with subjective experience (Fig. 4). Interestingly, we found a negative trend between change in power and how much participants paid attention to the feedback tone (slope = –0.130 ± 0.076, *t*_(23)_ = –1.703, *p* = 0.103; Fig. 4*A*); the results failed to reach significance only because of one outlier participant who completely ignored the tone; removing this participant from analysis yielded a slope of –0.261 ± 0.086, *t*_(22)_ = –3.036, *p* = 0.006) suggesting that participants who were actively attending to the feedback tone did not benefit from neurofeedback. Indeed, the participants who had the strongest effect of neurofeedback (participants 1–6, filled black triangles) were the ones who were neither fully attending nor fully ignoring the tone. There was, however, no such trend between alpha power and the participant’s self-reported level of relaxation (slope = –0.127 ± 0.189, *t*_(23)_ = –0.670, *p* = 0.510; Fig. 4*B*) or whether they were disturbed by the tone (slope = 0.077 ± 0.080, *t*_(23)_ = 0.964, *p* = 0.346; Fig. 4*C*). However, all the participants reported relaxation score of 5 and above (mean score, 7.958 ± 0.213), indicating that they felt relaxed after the neurofeedback task (Fig. 4*B*).

## Discussion

Using a novel design in which each participant was his/her own control, we tested whether participants were able to enhance their alpha power with valid neurofeedback more than false or no feedback. We found no enhancement of alpha power (during eyes closed state) over trials, although valid neurofeedback was provided during the major part of the experiment (33 out of the 60 trials; 50 s each). However, we found that in participants who could not sustain their alpha power, providing valid neurofeedback helped in sustaining alpha power within a trial more than when false or no feedback was provided. Surprisingly, participants who showed enhancement in alpha power with valid neurofeedback were the ones who did not pay too much attention to the neurofeedback tone. Overall, our results suggest that alpha neurofeedback, even when compared against a very stringent control condition, can help in the maintenance of the alpha power in some participants, and further recommend “passive attention” to the neurofeedback for best results.

### Comparison with previous studies

Our results are consistent with earlier studies where alpha enhancement beyond pre-training levels was not observed (Lynch and Paskewitz, 1971; Paskewitz and Orne, 1973; Lynch et al., 1974; Walsh, 1974; Plotkin, 1978, 1979; Cho et al., 2008; van Boxtel et al., 2012), and in contrast to studies which reported alpha enhancement (Hart, 1968; Kamiya, 1969; Brown, 1970). The temporal profile of alpha power, which tended to decrease with time, as well as the effect of neurofeedback, which helped to better maintain the power at elevated levels, are also in line with a previous study (Cho et al., 2008).

As discussed earlier, in our experimental design, the duration of feedback was short (50 s per trial), and valid feedback was inter-mixed with neutral and false feedback, to minimize the difference between valid and invalid conditions. We were especially concerned that prolonged invalid feedback might evoke a “surprise effect” because the feedback may be very different from what the participant may be feeling, or the participant may start ignoring the feedback if they realized that it was not valid or useful. Although such effects cannot be completely ruled out even in our design (in fact, in any neurofeedback design), having short duration of invalid feedback trials and inter-mixing these with a higher proportion of valid/neutral trials is likely to reduce these effects. Also, previous studies have typically used taped feedback from a different, control group of participants (Path et al., 1976; Watson and Herder, 1980; Hammond, 2005; Zoefel et al., 2011; Nan et al., 2012) or feedback based on a different frequency band (Egner et al., 2002; van Boxtel et al., 2012). The rate at which power varies in another individual or in a different frequency band is likely to be different, such that the statistics of the feedback signal (for example, how fast it varies with time) itself may be different across valid and invalid conditions, leading to a larger surprise effect. Further, it is possible that alpha power may depend on how the feedback tone varies over time (irrespective of the trial type), which may be different for valid versus invalid conditions in previous studies. In our study, these confounds are largely ruled out because the invalid feedback tone was based on the subjects’ own alpha power during a previous, valid trial. Therefore, the statistics of the tone signal was identical for valid versus invalid conditions. Indeed, when we asked the participants (after the completion of recording from all the participants) about the existence of the third (invalid) type of trials, all the participants who were reachable and remembered the experimental details (18 out of 24) were ignorant about the invalid trials (we did not ask the participants about the existence of the invalid trials immediately after their own recording because of the possibility of the inter-participant discussion about the experimental details). Further, keeping a short trial duration ensured that participants did not get drowsy or tired during the trial, which may also influence alpha power.

Although our design minimized the differences between valid and invalid conditions, the absolute effect of neurofeedback may be much smaller than previous studies For example, Ancoli and Kamiya (1978) suggested three critical factors to see positive effects of alpha feedback training, namely, (1) training for at least four sessions (2 h), (2) using continuous tone for feedback along with periodic scores of progress, and (3) using training trials with duration of at least 10 min. Thus, it is possible that more training sessions either on the same day or on different days could have led to long-term alpha enhancement, as observed in previous studies (van Boxtel et al., 2012; Dekker et al., 2014). Also, since we had a rather low ratio of valid to invalid trials (2:1), learning during training through operant conditioning may have been inefficient. In our design, the ratio was kept at 2:1 to have enough trials in each type (valid, invalid, and constant) to compare the effect of the neurofeedback training across type types. A lower proportion of invalid trials would have increased the duration of each experiment, which could have other disadvantages such as changes in the state of the subject due to drowsiness and fatigue, or changes in the impedance of the EEG electrodes. Further, as trials of different types were presented randomly, there might be a “carryover” effect of the training from one trial to the next. To reduce this effect, we provided sufficiently long inter-trial interval; participants were instructed to indicate when they wanted to start the next trial, such that a typical inter-trial interval was ∼10 s or more. Even within each trial, participants were asked to keep their eyes open for the first 15 s, and the main effect of feedback was studied only after that. However, any carryover training effect that is longer than the inter-trial duration may have influenced the power in the next trial, mixing the effects of valid and invalid feedback.

However, while these factors explain why the effect of neurofeedback was smaller than many previous studies, they cannot explain our main result, which is a significant difference in alpha power between valid and invalid conditions in almost half of the participants (25% after Bonferroni correction). Indeed, the main point here is not that the effect of neurofeedback was weak, but that there was a significant effect of neurofeedback despite several design limitations that were incorporated to keep the valid and invalid conditions as similar as possible.

One way to overcome the distortion in the learning dynamics because of invalid trials could be to use an alternate strategy in which subjects are asked to upregulate or downregulate their alpha power in different blocks of trials, while providing valid feedback in both conditions. To our knowledge, such a design has not been used yet, although both upregulation and downregulation have been studied in different experiments (van Boxtel et al., 2012; Ros et al., 2013). However, such a design need not necessarily provide the same type of control as the invalid trials in our design. For example, participants may use different strategies to control the alpha power in the up versus down regulation conditions, which might again affect the learning dynamics for either type of task. In case there is a difference in alpha power in upregulation and downregulation conditions, it could be due to a subjects’ ability to suppress alpha in downregulation condition instead of enhancement in the upregulation condition. So, if we are specifically interested in whether alpha can be voluntarily enhanced by valid feedback, an invalid feedback (unknown to the subject) may provide a more direct control. Further, this invalid feedback condition has been used in many earlier studies (albeit always on a different control group of participants), so our design follows a popular, well studied paradigm. This alternate strategy of interleaved blocks of upregulation and downregulation, nonetheless, can be used in future studies to provide a different type of control that can complement the control used in this study.

### Neurofeedback and attention

Unlike previous studies where participants were asked to focus on the tone to control their alpha activity (Kamiya, 1969; Nowlis and Kamiya, 1970; Plotkin, 1978), in our study the participants were completely free to ignore the tone if the tone was distracting or did not aid in improving their performance score. We found that participants, who used the feedback tone for getting information about their alpha power without focusing too much to it, were the ones who could successfully maintain their alpha level. This is consistent with previous reports that have shown that attending to a stimulus leads to reduction in alpha power (Kelly et al., 2006b; Klimesch et al., 2007; Sadaghiani et al., 2010; Händel et al., 2011). Similarly, it is likely that attending to the neurofeedback tone may have reduced alpha power.

In our study, the main analysis was performed when the subjects had their eyes closed. This also may have contributed to the small effect of neurofeedback, since alpha power is much stronger when eyes are closed and may have reached some sort of “saturation level.” Indeed, some previous studies have shown larger alpha enhancement when eyes were kept open (Brown, 1970; Zoefel et al., 2011; van Boxtel et al., 2012), even with no or invalid feedback (Ros et al., 2013; Enriquez-Geppert et al., 2014; Jurewicz et al., 2018). Even in our results, a weak but significant long-term enhancement in alpha power was indeed observed during the eye open condition. We preferred the eye closed state because this condition has fewer confounding variables, such as saccadic eye movements, which are known to modulate the power and phase in many frequency bands, including alpha (Bartlett et al., 2011; Staudigl et al., 2017). As before, while the eyes closed state may have resulted in a smaller overall effect of neurofeedback, this cannot explain the difference in alpha power between the valid and invalid conditions that we observed.

As opposed to the testing (neurofeedback) period, the baseline power used for calibration was recorded when the eyes were open (during the calibration period). This was done because we were particularly interested in quantifying the effect of neurofeedback in enhancing alpha activity from a baseline level where least amount of alpha activity was present. Furthermore, taking baseline measurement during eyes open state allowed the participants to feel a “definitive signal” when they closed their eyes since the tone frequency increased instantaneously. Comparison of alpha in eye open versus eye closed conditions is complicated, since different subjects might have different levels of alpha synchronization on eyes closure which may not be directly related to the eyes open state. In our case, this issue is of less relevance because our main comparison (between the valid and invalid conditions) is always during eye closed state only. Note that taking baseline measurement with eyes closed condition only changes Pc in Equation 1, which only shifts the operating frequency of the feedback tone away from 1000 Hz (Eqs. 1, 2) without changing any other dynamics.

Another potential confound could be related to the use of FCz as a reference electrode, since this electrode is near the C3 and C4 electrode positions where mu rhythms (which approximately have the same frequency range as alpha) might be predominant (Gastaut et al., 1954). However, mu rhythms are typically associated with planning of motor movements (Pfurtscheller and Neuper, 1997), but our participants were not engaged in any type of motor activity. Thus, it is unlikely that the choice of the reference electrode affected our results.

### Mechanisms of alpha neurofeedback

Our results show a short-term benefit of valid neurofeedback in alpha power maintenance that does not translate to any long-term benefits. Neural mechanisms behind such short-term effects are unclear. Recent EEG-fMRI studies have demonstrated that neurofeedback can lead to a plastic increase in the connectivity within the salience network, which was detectable several minutes after the termination of training (Ros et al., 2013). Further, the increase in salience (default-mode) network connectivity was negatively (positively) correlated with changes in “on task” mind-wandering as well as resting state alpha rhythm (Ros et al., 2013). Default mode network, which primarily consists of the ventral medial prefrontal cortex (vmPFC) and posterior cingulate cortex (PCC), shows significant activity while individuals are not engaged in the external environment and are at a resting-state condition (Buckner et al., 2008; Uddin et al., 2009). On the other hand, salience network of the brain is active during the performance of sensory attention task (Sadaghiani et al., 2010). Neurofeedback can also change dynamic resonant loops in the cortical and thalamocortical circuit (Lubar, 1997), potentially by changing their excitability (Ros et al., 2010). Unfortunately, because we only recorded from occipital electrodes, we cannot study the interaction between visual and default mode or salience networks or measure changes in the excitability of different brain structures. Even if we had coverage of the entire brain, significant volume conduction and poor source localization with EEG (Nunez et al., 1997; Nunez and Srinivasan, 2006) would likely have made analysis and interpretation of data difficult. Simultaneous fMRI-EEG recording while providing neurofeedback, similar to the study by Ros et al. (2013), may be needed to better understand the effect of neurofeedback at short time scales.

Behaviorally, neurofeedback training was perceived relaxing by almost all the participants, with an average subjective relaxation score of 7.96 ± 0.21 out of 10, consistent with previous studies (Kamiya, 1969; Brown, 1970; Nowlis and Kamiya, 1970), although there was no increase in alpha power over trials and the change in alpha power between valid and invalid trials was uncorrelated with the relaxation score (Fig. 4*B*). Instead, this relaxation may be attributed to various factors described by Plotkin, which include sensory deprivation, sustained alertness, concentration/meditation, introspective sensitization, expectation, perceived success at the feedback task due to the isolated setting during the neurofeedback training (Plotkin, 1978, 1979). Thus, while we show that neurofeedback indeed leads to an increase in alpha power even under the most stringent control conditions and show that this works best for subjects who otherwise cannot sustain their alpha power and when they attend to the feedback “passively” (that is, have intermediate attention scores), we do not comment on its potential beneficial physiological effects. Since such different types of feedback are inter-mixed in our design, such questions are beyond the scope of our study.

## Synthesis

Reviewing Editor: Bradley Postle, University of Wisconsin Decisions are customarily a result of the Reviewing Editor and the peer reviewers coming together and discussing their recommendations until a consensus is reached. When revisions are invited, a fact-based synthesis statement explaining their decision and outlining what is needed to prepare a revision will be listed below. The following reviewer(s) agreed to reveal their identity: Katarzyna Paluch, Tomas Ros.

Reviewer #1

My main concern regarding the presented work is following: was with used training design efficient operant conditioning indeed possible? If valid and invalid trials are presented to the same participant, the valid condition has to markedly outnumber the invalid one, otherwise it will be ignored and the learning will never occur. In the presented study participants were not informed by the experimentators about existence of invalid condition thus, subjectively the trial composition was 75% valid to 25% invalid. However, the real proportion was 50% valid to 25% invalid to 25% constant (which is worryingly low).

I agree with the authors that within-subjects training design has some advantages that cannot be accomplished in between-subjects studies. However my recommendation would be the usage of intermixed trails of up-regulation and down-regulation of alpha band (preceded with appropriate instructions). Such design will provide the same type of control without distorting learning dynamics in the way that might happened in case of invalid trials. Finally, if I understood correctly, subjects were aware that during constant trials the tone is non-informative. Figure 1E, however, shows that invalid and constant trials are more alike than valid ones while Figure 2 shows that this is true only for the subgroup of subjects, while in the rest of them all three types of trials are similar. Is it possible that participants who behaved differently in invalid/constant vs neutral condition became aware of the existence of invalid condition and were able to correctly recognize it during sessions? Is it possible that decrease of alpha power in passive condition and invalid trials indicates increasing drowsiness, which was prevented in valid trials by the active effort of participants or attention to the tone. In my opinion the probability that they became aware of them in the course of experiment can't be ruled out, unless they were asked about it directly at the end of the study and denied it. Authors should address this issue and if possible provide some evidence that it was not the case or at least elaborate on it the discussion.

INTRODUCTION:

•It draw my attention that majority of the studies cited in the introduction are quite old (from 70's), while alpha band is one of the most often studied bands in the context of EEG-NFT and there are many more recent works. However, I admit that in case of NFT earlier works are often more conclusive and better designed that the once conducted during the time of the highest interest in this method. It was my impression that the introduction would be more comprehensive if some more recent works were included by the authors.

METHODS:

• Why the questionnaires were provided only at the end of the entire study and not after completion of each session or if possible each trail? - In my opinion single evaluation might be highly biased by the most recent and thus vivid experience of the last session.

• Was the sequence of valid / invalid trials randomised between subjects?

•I really appreciated the detailed description of the method that was provided by the Authors however to ensure replicability I would recommend adding few more details of EEG-NFT procedure: application of high and low-pass filters (and their parameters) for online data processing; overlap between subsequent FFT windows; delay with which feedback was provided to the participants

•I would recommend to add also information about artefacts rejection / control both muscular and eye movement / blinks

•Lines 200-201 are unclear for me, they suggest that the baseline was the same for subsequent sessions while I think, that what authors meant was that it was the same for all trials within one session?

•In line 53 there is one excessive bracket, in the text “MATLAB” is written inconsistently either in upper or lower-case.

DISCUSSION:

•How Authors interpret the decrease of alpha power with eyes closed during constant trials could it be due to the increasing drowsiness in this inactive condition? When subjects kept their eyes open, the power of alpha band usually increase in time, even when no feedback is provided (e.g. Enriquez-Geppert et al., 2014; Jurewicz et al., 2018; Ros et al., 2013)

Dempster, T., Vernon, D., 2009. Identifying indices of learning for alpha neurofeedback training.

Enriquez-Geppert, S., Huster, R.J., Scharfenort, R., Mokom, Z.N., Zimmermann, J., Herrmann, C.S., 2014. Modulation of frontal-midline theta by neurofeedback.

Ros, T., Théberge, J., Frewen, P.A., Kluetsch, R., Densmore, M., Calhoun, V.D., Lanius, R.A., 2013. Mind over chatter: plastic up-regulation of the fMRI salience network directly after EEG neurofeedback.

Jurewicz K., Paluch K., Kublik E., Rogala J., Mikicin M., Wróbel A. (2018) EEG-neurofeedback training of beta band (12-22 Hz) affects alpha and beta frequencies - A controlled study of healthy population.

Reviewer #2

This is a rigorously designed study which provides important evidence for a neurophysiologically-specific effect of EEG-based neurofeedback. This is done by contrasting alpha rhythm self-regulation between valid and invalid neurofeedback trials. Such experimental evidence is vital in light of current controversies on whether neurofeedback has a tangible and neurophysiologically-specific effect on brain activity, or whether its effects are a consequence of mere placebo. For example, see the following reference for a sceptical view

Thibault RT, Lifshitz M, Raz A. The self-regulating brain and neurofeedback:

Experimental science and clinical promise. Cortex. 2016 Jan;74:247-61.

doi: 10.1016/j.cortex.2015.10.024.

Overall, I have no problems with the design, implementation and analysis of results, which appear competent. Please see below for several comments that may improve the quality and readability of the manuscript.

Why was data from the first session discarded?

Why was the baseline alpha power calculated under an eyes-open condition while the neurofeedback training was done under eyes-closed? Different subjects might have different levels of alpha synchronisation upon eyes closure which are not directly related to the eyes open state. This limitation should perhaps be acknowledged in the Discussion

Why was Fcz used as the reference electrode? Have the authors made sure that this region is more “alpha-neutral” than an earlobe reference? Due to volume conduction, mu-rhythms from the motor cortex may have indirectly contaminated this reference. A limitation to acknowledge in my opinion.

“was” typo on line 175

“ABSTRACT: We found that neurofeedback did not enhance alpha activity beyond pre-trained state within our experimental duration.”

I think the results are more subtle than inferred by this statement.

Firstly, the pre-trained state was recorded under eyes open, but the training was conducted under eyes closed. Secondly, the results of the eyes open suggest the slope was significantly greater than zero (N=24, t (23) =2.936, p=0.007). So perhaps it is better to break-down the effect into eyes open and eyes closed separately in the abstract (as well as mention the eyes closed level probably reflected saturation of alpha rhythmicity )

“For all trial types, a peak was observed as soon as this participant closed the eyes which may have some contribution from muscle artefact associated with eye closing.” This is more likely due to the “alpha squeak” effect upom eye closure rather than muscle artifact. (Storm van Leeuwen W., Kemp, A., Kniper, J. (1960): Concerning the Squeak' phenomenon of the alpha rhythm. Electroencephalogr. Clin. Neurophysiol. 12,244.)

Dicrepancy between slope significance (eyes open and closed) on line 216 vs line 222?

“Therefore, there was no benefit of neurofeedback over the entire course of the experiment.” NOT TRUE For t-test for comparison on line 238?

## Author Response

Reviewer #1

My main concern regarding the presented work is following: was with used training design efficient operant conditioning indeed possible? If valid and invalid trials are presented to the same participant, the valid condition has to markedly outnumber the invalid one, otherwise it will be ignored and the learning will never occur. In the presented study participants were not informed by the experimentators about existence of invalid condition thus, subjectively the trial composition was 75% valid to 25% invalid. However, the real proportion was 50% valid to 25% invalid to 25% constant (which is worryingly low).

We completely agree with the reviewer that since we had a rather low ratio of valid to invalid trials (2:1), learning during training through operant conditioning might not be efficient. This could be one of many reasons why the effect of neurofeedback in our study was rather small. However, while this (and other) factors explain why the effect of neurofeedback was smaller than many previous studies, the main point of the paper is that a significant difference in alpha power between valid and invalid conditions can be observed in almost half of the participants (25% after Bonferroni correction) in spite of these limitations. We had discussed several such limitations in the “Comparison with previous studies” section of the Discussion, but this issue was not discussed. We thank the reviewer for pointing this out, and we now discuss this issue in the same section of the paper.

Unfortunately, it is difficult to address this concern effectively in our design. The ratio was kept at 2:1 to have enough trials in each type (valid, invalid and constant) to compare the effect of the neurofeedback training across trial types. While we agree that it would have been better to have a lower percentage of invalid trials, we would have needed a larger number of trials to have enough invalid trials for proper comparison, which would have increased the duration of the experiment. This would have other disadvantages, such as changes in the state of the subject due to drowsiness or fatigue, or changes in electrode impedance. We now discuss these points in the Discussion.

I agree with the authors that within-subjects training design has some advantages that cannot be accomplished in between-subjects studies. However my recommendation would be the usage of intermixed trails of up-regulation and down-regulation of alpha band (preceded with appropriate instructions). Such design will provide the same type of control without distorting learning dynamics in the way that might happened in case of invalid trials.

We thank the reviewer for the recommendation. Indeed, in a few recent reports, researchers have studied up-regulation as well as down-regulation of alpha rhythm in different experiments. As the feedback is contingent in both type of trials, we agree that this would not distort the learning dynamics in the way that might happen in case of invalid trials. To the best of our knowledge, a design consisting of intermixed blocks of up-regulation and down-regulation of alpha has not been used and would indeed be a very interesting design. We can compare alpha power in blocks of up-regulation versus down-regulation in the same subject and test for differences in alpha power in the two conditions.

However, such a design need not necessarily provide the same type of control as the invalid control trials in our design. For example, participants may use different strategies to control the alpha-power in the different type of trials, which might again affect the learning dynamics for either type of task. In case there is a difference in alpha power in up- and down-regulation conditions, it could be due to a subjects' ability to suppress alpha in down-regulation condition instead of enhancement in the up-regulation condition. Since we were specifically interested in whether alpha can be voluntarily enhanced by proper feedback, we have used an invalid feedback condition (unknown to the subject) which may provide a more direct control. Further, this invalid feedback condition has been used in many earlier studies (however always on a different “control) group of participants), so our design build on a popular, well studied paradigm. A paragraph has now been included in the Discussion section (last paragraph of the “Comparison with previous studies” section) to highlight the above point.

Finally, if I understood correctly, subjects were aware that during constant trials the tone is non-informative. Figure 1E, however, shows that invalid and constant trials are more alike than valid ones while Figure 2 shows that this is true only for the subgroup of subjects, while in the rest of them all three types of trials are similar.

We thank the reviewer for bringing out this interesting observation. Indeed, even though the invalid trials were matched to the valid ones in stimulus statistics and behavioral state, the invalid alpha power was actually matched to the constant condition for many subjects. This may mean that alpha dynamics actually reflect the potency/validity/usefulness of the feedback, since on average, both invalid and constant feedback are equally useless/uninformative. We have now elaborated this in the Results section.

Is it possible that participants who behaved differently in invalid/constant vs neutral condition became aware of the existence of invalid condition and were able to correctly recognize it during sessions? Is it possible that decrease of alpha power in passive condition and invalid trials indicates increasing drowsiness, which was prevented in valid trials by the active effort of participants or attention to the tone. In my opinion the probability that they became aware of them in the course of experiment can't be ruled out, unless they were asked about it directly at the end of the study and denied it. Authors should address this issue and if possible provide some evidence that it was not the case or at least elaborate on it the discussion.

We thank the reviewer for this valuable comment. To address this, we asked the participants whether they were aware of the third (invalid) type of trials during the experiment. The participants who were reachable and remembered the experimental details (18 out of 24) replied remembering only two types of trials: valid and constant and were completely ignorant about the third type trial (invalid). We now discuss this point in the Discussion (second paragraph of the “Comparison with previous studies” section).

INTRODUCTION:

It draw my attention that majority of the studies cited in the introduction are quite old (from 70's), while alpha band is one of the most often studied bands in the context of EEG-NFT and there are many more recent works. However, I admit that in case of NFT earlier works are often more conclusive and better designed that the once conducted during the time of the highest interest in this method. It was my impression that the introduction would be more comprehensive if some more recent works were included by the authors.

We thank the reviewer for the suggestion. Few recent works have now been cited in the introduction (Dekker et al., 2014; Dempster and Vernon, 2009; Ros et al., 2013). More works, if suggested, would be added to the article. It was rightly noted by the reviewer that more conclusive work was done during the highest interest in the method. Also, question pertaining to enhancement of alpha-rhythm was also studied during this period; recent works had mainly used this paradigm to look at other behavioral and mental aspects of the training.

METHODS:

Why the questionnaires were provided only at the end of the entire study and not after completion of each session if possible each trial? - In my opinion single evaluation might be highly biased by the most recent and thus vivid experience of the last session.

We agree with the reviewer that having a questionnaire at the end of each session or perhaps each trial could have overcome the bias. However, we were worried that answering questions after each session (or trial) could have disrupted the continuity of the training and potentially could have caused disruption in the learning and also increased the duration of the experiment. Also, we were more interested in the overall effect of neurofeedback that was experienced for the entire duration of the study. Two lines have been added to the Methods section to discuss this point.

Was the sequence of valid / invalid trials randomised between subjects?

Yes, the sequence of valid and invalid trials was randomized between subjects. We now have mentioned that in the Methods section.

I really appreciated the detailed description of the method that was provided by the Authors however to ensure replicability I would recommend adding few more details of EEG-NFT procedure: application of high and low-pass filters (and their parameters) for online data processing; overlap between subsequent FFT windows; delay with which feedback was provided to the participants.

For estimating alpha power, we have used multi-taper method at each second of data. So, there was no overlap of subsequent windows. The delay was not measured explicitly but was negligible since the feedback tone sounded almost continuous. This point is now elaborated in the Methods section.

I would recommend to add also information about artefacts rejection / control both muscular and eye movement / blinks

In our paradigm, we did not have online artifact rejection. However, during calibration, in case of any eye movement/blink (assessed manually by observing the time-frequency power spectra), we repeated the trial. During actual task, as the eyes were closed and subjects were calmly sitting in the chair, we did not observe any significant artefacts due to either muscular or eye movement. We now have included one line in the Methods section of the manuscript stating above.

Lines 200-201 are unclear for me, they suggest that the baseline was the same for subsequent sessions while I think, that what authors meant was that it was the same for all trials within one session?

Thanks for pointing this out. That is right. The baseline measure was same for all the trials of the same session. We now have corrected this line.

In line 53 there is one excessive bracket, in the text “MATLAB” is written inconsistently either in upper or lower-case.

Thanks for pointing this out. This has been corrected now.

DISCUSSION:

How Authors interpret the decrease of alpha power with eyes closed during constant trials could it be due to the increasing drowsiness in this inactive condition? When subjects kept their eyes open, the power of alpha band usually increase in time, even when no feedback is provided (e.g. Enriquez-Geppert et al., 2014; Jurewicz et al., 2018; Ros et al., 2013)

We agree that power of the alpha power can increase with time during eyes open condition, even when no alpha contingent feedback is provided as shown in earlier reports. Even we have observed that alpha power increased during eyes-open condition during which no feedback was provided. The second reviewer also pointed out that our results are limited to eyes closed condition only. We have now described, at several places, that our results are only valid for eye closed condition, and that alpha power can actually increase over time even without feedback (second paragraph of the “Neurofeedback and Attention” section).

Reviewer #2

This is a rigorously designed study which provides important evidence for a neurophysiologically-specific effect of EEG-based neurofeedback. This is done by contrasting alpha rhythm self-regulation between valid and invalid neurofeedback trials. Such experimental evidence is vital in light of current controversies on whether neurofeedback has a tangible and neurophysiologically-specific effect on brain activity, or whether its effects are a consequence of mere placebo. For example, see the following reference for a sceptical view

Thibault RT, Lifshitz M, Raz A. The self-regulating brain and neurofeedback:

Experimental science and clinical promise. Cortex. 2016 Jan;74:247-61. doi: 10.1016/j.cortex.2015.10.024.

We thank the reviewer for the appreciative remarks.

Overall, I have no problems with the design, implementation and analysis of results, which appear competent. Please see below for several comments that may improve the quality and readability of the manuscript.

Why was data from the first session discarded?

The data from the first session was not used for analysis because in these trials participants were getting accustomed with the task itself (pre-training phase). Further, in this session we had only two types of trials: valid and constant, which was different from the rest of the experimental sessions in which we had all the three trial types. This has been now clarified in the Methods section of the article. Importantly, including the first session data for the analysis does not change the main result (please refer to the figure below where data from all the sessions were used for the analysis). One line stating the above has been included in the Methods section.

Figure 1: Same as Figure 2 of the main text, but after including data from the first session. Difference in alpha power between valid and invalid conditions is significant for 10 subjects (7 after Bonferroni correction), as opposed to 11 subjects (6 after Bonferroni correction) before.

Why was the baseline alpha power calculated under an eyes-open condition while the neurofeedback training was done under eyes-closed? Different subjects might have different levels of alpha synchronisation upon eyes closure which are not directly related to the eyes open state. This limitation should perhaps be acknowledged in the Discussion

We agree that different participants might have different levels of alpha synchronization upon eyes closure which are not directly related to the eyes open state. We had earlier briefly discussed this in the third paragraph of “Neurofeedback and attention” section of the Discussion, but we have now expanded the discussion to specifically include these points.

Why was Fcz used as the reference electrode? Have the authors made sure that this region is more “alpha-neutral” than an earlobe reference? Due to volume conduction, mu-rhythms from the motor cortex may have indirectly contaminated this reference. A limitation to acknowledge in my opinion.

We thank the reviewer for the comment. Based on a similar comment in a previous submission, we had already discussed this issue in the fourth paragraph of the “Neurofeedback and attention” section of the Discussion.

“was” typo on line 175

The typo was corrected.

“ABSTRACT: We found that neurofeedback did not enhance alpha activity beyond pre-trained state within our experimental duration.” I think the results are more subtle than inferred by this statement. Firstly, the pre-trained state was recorded under eyes open, but the training was conducted under eyes closed. Secondly, the results of the eyes open suggest the slope was significantly greater than zero (N=24, t (23) =2.936, p=0.007). So perhaps it is better to break-down the effect into eyes open and eyes closed separately in the abstract (as well as mention the eyes closed level probably reflected saturation of alpha rhythmicity).

We agree and have changed the Abstract to state that no enhancement in alpha power was observed only during eye closed state, and it probably is due to saturation of alpha rhythmicity. However, we could not add the eye open condition because we are already at the word limit, and the eye open state was not as well controlled (as discussed in the Results section). We have now added a line in the Discussion (second paragraph of the “Neurofeedback and attention” section) emphasizing that alpha enhancement was indeed observed during eye open state.

“For all trial types, a peak was observed as soon as this participant closed the eyes which may have some contribution from muscle artefact associated with eye closing.” This is more likely due to the “alpha squeak” effect upom eye closure rather than muscle artifact. (Storm van Leeuwen W., Kemp, A., Kniper, J. (1960): Concerning the Squeak' phenomenon of the alpha rhythm. Electroencephalogr. Clin. Neurophysiol. 12,244.)

We thank the reviewer for pointing this out. This was included in the Result section and is highlighted in red font.

Discrepancy between slope significance (eyes open and closed) on line 216 vs line 222?

This apparent discrepancy is only because the first slope value (line 216) is for all the 24 participants, while the second analysis is for a representative subject. We have now added “for this subject” to clarify this now.

“Therefore, there was no benefit of neurofeedback over the entire course of the experiment.” NOT TRUE For t-test for comparison on line 238?

Thanks for pointing this out. We have now qualified that this holds only for eye closed condition.

References:

Dekker MKJ, Sitskoorn MM, Denissen AJM, van Boxtel GJM (2014) The time-course of alpha neurofeedback training effects in healthy participants. Biological Psychology, SAN (Society of Applied Neuroscience) Special issue on Neurofeedback 95:70-73.

Dempster T, Vernon D (2009) Identifying Indices of Learning for Alpha Neurofeedback Training. Appl Psychophysiol Biofeedback 34:309.

Ros T, Théberge J, Frewen PA, Kluetsch R, Densmore M, Calhoun VD, Lanius RA (2013) Mind over chatter: Plastic up-regulation of the fMRI salience network directly after EEG neurofeedback. NeuroImage 65:324-335.
